# HR factors affecting the availability of medical products in developing countries: a systematic literature review

**DOI:** 10.1186/2052-3211-7-S1-P3

**Published:** 2014-12-17

**Authors:** Pamela Steele

**Affiliations:** 1 Pamela Steele Associates (PSA) Ltd, Oxford, UK

## Background

Developing countries face many complex challenges in the provision of essential medicines. The objective of this review was to establish what the existing literature says about in-country, public health supply chain factors which affect the availability of medicine at the point of service delivery in developing countries.

## Method

A systematic literature review methodology was adopted to find, evaluate, analyse, and synthesize literature in a transparent, replicable manner. Retrieved articles were categorized by the following topics: year of publication, journal name, whether from practice or academia, research methods employed, and country of residence of corresponding author, in order to establish trends in publications from 1970 to date. A series of keyword searches were conducted on electronic databases between May 2012 and July 2012. The literature obtained was evaluated against specified criteria for relevance and quality. Reference lists from articles that met the selection criteria were used to locate further literature; grey literature from other sources was also assessed against the specified criteria for relevance and quality.

## Results

The importance of the role of supply chain is clearly established. The study identified a number of factors that affect medicine availability at service delivery points in developing countries and proposes a set of propositions that can be used for empirical investigation. Although effective SCM requires a focus on supply chain functions, Human

Resources (HR) is a cross-cutting issue, touching every function in the supply chain from quantification to service delivery. Hr It was found to be one of the challenges to ensuring medicine availability.

## Discussion

Peer-reviewed publications on factors affecting medicine availability are few, and many of those reviewed focused on other thematic areas such as the insurance, financing, affordability, regulations, selection, and rational use of medicine, as well as the health workforce and intellectual property rights. Less than half of all publications were written by authors residing in developing countries. While it was possible to identify the factors in the thematic analysis the study did not fully investigate them and the scale of the impact on availability by different factors. Further research is needed to determine this.

## Lessons learned

This is the first attempt to relate supply chain to World Health Organization (WHO) health systems strengthening building blocks and is the most comprehensive presentation of public health supply chain literature.

